# APC/C and retinoblastoma interaction: cross-talk of retinoblastoma protein with the ubiquitin proteasome pathway

**DOI:** 10.1042/BSR20160152

**Published:** 2016-09-16

**Authors:** Ajeena Ramanujan, Swati Tiwari

**Affiliations:** * School of Biotechnology, Jawaharlal Nehru University, New Mehrauli Road, New Delhi 110067, India

**Keywords:** anaphase promoting complex/cyclosome, cell cycle, FZR1, human papilloma virus, LxCxE, retinoblastoma

## Abstract

The ubiquitin (Ub) ligase anaphase promoting complex/cyclosome (APC/C) and the tumour suppressor retinoblastoma protein (pRB) play key roles in cell cycle regulation. APC/C is a critical regulator of mitosis and G_1_-phase of the cell cycle whereas pRB keeps a check on proliferation by inhibiting transition to the S-phase. APC/C and pRB interact with each other via the co-activator of APC/C, FZR1, providing an alternative pathway of regulation of G_1_ to S transition by pRB using a post-translational mechanism. Both pRB and FZR1 have complex roles and are implicated not only in regulation of cell proliferation but also in differentiation, quiescence, apoptosis, maintenance of chromosomal integrity and metabolism. Both are also targeted by transforming viruses. We discuss recent advances in our understanding of the involvement of APC/C and pRB in cell cycle based decisions and how these insights will be useful for development of anti-cancer and anti-viral drugs.

## INTRODUCTION

**Figure 1:**
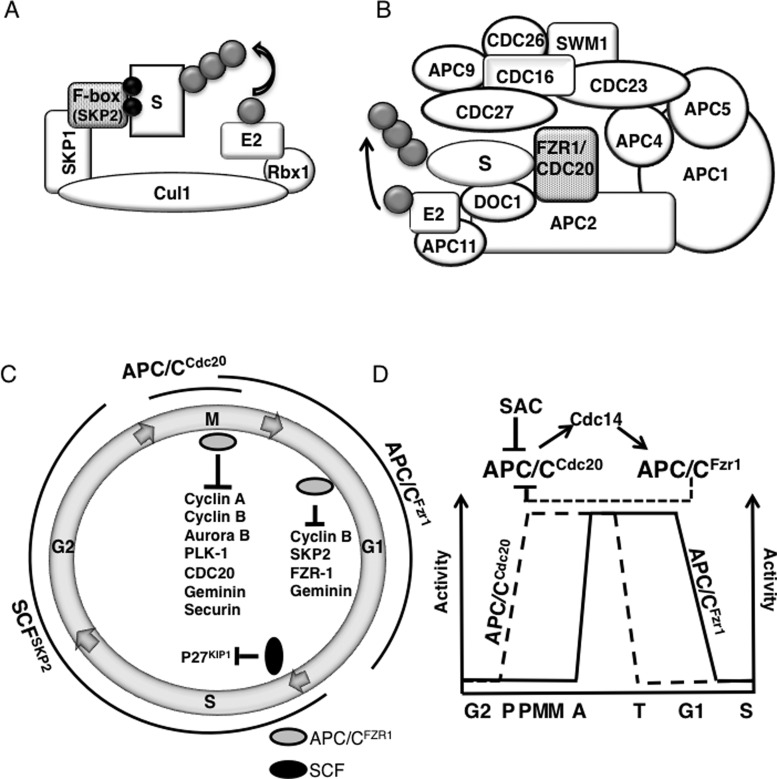
Regulation of cell cycle by the SCF and APC/C E3 ligases Schematic diagrams of the modular structure of the SCF (**A**) and APC/C (**B**) showing the relative positions of various subunits. The stages of the cell cycle where activities of these complexes regulates key events are shown in (**C**) along with some of the key substrates. The activities of two APC/C assemblies (APC/C^CDC20^ and APC/C^FZR1^) are regulated in opposite manner by phosphorylation resulting their manifestation at different stages (**D**). APC/C^CDC20^ is activated upon phosphorylation by the mitotic cyclin/CDK complex but its activity is kept in check by the SAC. Once SAC is satisfied, APC/C^CDC20^ targets mitotic cyclins resulting in decrease in kinase activity thus inactivating APC/C^CDC20^. At the same time, activation of the phosphatase Cdc14 dephosphorylate FZR1 resulting in activation of APC/C^FZR1^.

Somatic cell cycle has alternating DNA synthetic (S) and mitotic (M) phases, separated by gap phases (G_1_ and G_2_). The correct sequence of events, a robust feature in cell cycle, is maintained by timely degradation of cell-cycle regulators by ubiquitin proteasome pathway (UPP). The UPP consists of the ubiquitin (Ub)-activating enzyme (E1), Ub-conjugating enzyme (E2) and Ub ligases (E3) that covalently link Ub on to target proteins either singly or in chains that are formed using various internal lysines of Ub. Although chains linked through Lys-48 and Lys-11 lead to destruction of substrate proteins by 26S proteasome, monoubiquitination and chains other than Lys-48 and Lys-11 linkages have non-proteolytic functions. Two related multi-subunit E3s, the anaphase promoting complex/cyclosome (APC/C) and the Skp1/Cul1/F-box (SCF) complex are crucial for timely proteolysis of cell cycle proteins (Figure 1A). Although SCF performs throughout the cell cycle, APC/C remains active from M to late G_1_ [1]. The APC/C has emerged as a critical regulator of mitosis not only due to its role in spindle assembly checkpoint (SAC) but also for being crucial for degradation of mitotic cyclins and securin that paves the way for completion of mitosis. On the other hand, it is equally important for post-mitotic decisions of the cell about proliferation, differentiation and quiescence. Other than cell cycle, the APC/C also regulates neuronal development and metabolism [2–4]. With 15 subunits in vertebrates, APC/C is one of the largest and the most complex E3s known to date and has been a subject of intense investigation due to its wide-ranging roles. Combined use of cryo-electron microscopy, mass-spectroscopy and docking of crystal structures and homology models allowed reconstruction of the pseudo atomic model and later with more advanced cryo-EM technology, of the atomic scale structure of its co-activator in complex with the E2s, UbcH10 and Ube2S and with one of its inhibitory protein, Emi1 [5–7]. These studies have given an insight into the mechanism of initiation of ubiquitination, inhibition of the complex and regulation by co-activators. However, we are still far from a complete mechanistic understanding of its various functions, its complete interactome and substrates and its regulation by phosphorylation. This review will focus on G_1_-S regulation by APC/C and the readers are directed to other excellent recent reviews on its other functions [1,8–10]. We review the recent advances in our understanding of how APC/C regulates G_0_/G_1_ stage and controls S-phase entry and discuss the implications of its interaction with the tumour suppressor protein retinoblastoma (pRB) for cell cycle regulation and development of anti-viral and anti-cancer drugs.

## G_1_-S REGULATION BY APC/C

CDC20 and FZR1 are two related co-activators that recruit substrates to APC/C in two distinct assemblies, APC/C^CDC20^ and APC/C^FZR1^ [11]. Both CDC20 and FZR1 are structurally related and consist primarily of a seven-bladed WD40-repeat-propeller that facilitates protein–protein interactions. The CDC20 and FZR1 bound forms of APC/C demonstrate both different and overlapping substrate specificities [12,13]. The most well characterized degrons in APC/C substrates are the destruction box (D-box) [14] and the KEN-box [15], but other targeting motifs have also been identified [16–18]. CDC20 and FZR1 recognize specific target proteins depending on the recognition sequences present, such as the destruction box (D-box) for CDC20, and the D-box, KEN box and CRY box for FZR1 [19,20].

APC/C^CDC20^ is activated upon phosphorylation by mitotic cyclin-CDK but its activity is kept in check till the SAC is satisfied with CDC20 as a component of the mitotic checkpoint complex [21]. Once SAC is satisfied APC/C^CDC20^ activity is unleashed resulting in ubiquitination and destruction of many substrates. Critical among these are the mitotic cyclins and securin (Figure 1B). This leads to low kinase activity and inactivation of APC/C^CDC20^ during metaphase. Low kinase activity also results in dephosphorylation of FZR1 and activation of APC/C^FZR1^ that has a much broader substrate range. It also targets CDC20 and any remaining mitotic cyclin and some other mitotic substrates for degradation [22]. APC/C^FZR1^ continues working till late G_1_ and negatively regulates DNA replication and cell proliferation through degradation of multiple proteins that helps in maintenance of G_1_ state (Table 1) [10,23,24]. Some of these critical targets include pro-proliferative proteins like Polo-like kinase 1 (PLK1), Aurora kinase A and CDC25A, the activator of CDK1 and proteins required for DNA replication (e.g. geminin, Cdc6 and TK1). SKP2, a subunit of the SCF E3 is an important target of FZR1 that results in stabilization of CKI protein p27^KIP1^, further stabilizing the G_1_ state. Another critical target of APC/C^FZR1^ in maintaining G_1_ is the transcription factor E2F1 that promotes the S-phase genes once it is released by phosphorylation of pRB [25]. E2F1 is targeted for degradation by APC/C^FZR1^ until the G_1_/S transition. Hence APC/C^FZR1^ on one hand maintains low levels of E2F1 to inhibit S-phase; on the other it engages with pRB in mediating APC/C^FZR1^ dependent degradation of SKP2 and allowing the build-up of CKI proteins to inhibit S-phase, thus utilizing a two pronged strategy involving transcriptional and post-translational mechanisms to prevent cells from entering the S-phase.

**Table 1:** Substrates of APC/C^FZR1^

G_1_	Mitosis	Reference
FZR1		[27]
SKP2		[132]
Cyclin B1	Cyclin B1	[133,134]
FoxM1		[135]
CDCA3		[136]
Anillin		[137]
Nek2		[15]
B99		[15]
E2F1		[25]
TOME1		[138]
Geminin	Geminin	[139]
	Aurora B	[140]
	CDC6	[141]
	CKAP2	[142]
	CDC20	[143]
	Cyclin A	[144]
	ETS2	[145]
	Claspin	[46]
	Id2	[146]
	PLK1	[147]
	Rcs1	[148]
	Securin	[149]
	Sgo1	[150]
	SnoN	[151]
	Tpx2	[152]
	Xkid	[153]
	CLB2	[154]
	CDC5	[155]
	HSL1	[155]
	CDC25A	[138]
	NDD1	[156]
	CtIP	[157]

### Regulation of FZR1

For cells to enter the S-phase, the activity of APC/C^FZR1^ has to be brought down in a regulated manner. There are several mechanisms that regulate FZR1 levels in cells that allow APC/C to be shut-off in late G_1_. Although FZR1 RNA levels remain constant throughout the cell cycle, its protein levels fluctuate. FZR1 levels are high in mitosis, but lowered in late G_1_- and S-phases [26]. FZR1 mediates its own degradation in late G_1_. This process of self-destruction requires the two D-boxes of FZR1 [27]. Another mechanism of APC/C^FZR1^ inactivation is the ubiquitination of APC/C-specific E2 UBCH10 by APC/C^FZR1^ itself, thereby providing a negative feedback mechanism [28,29]. Phosphorylation also regulates FZR1. The binding of FZR1 to APC/C depends on FZR1 phosphorylation status. CDC28 mediated phosphorylation of FZR1 excludes it from the nucleus and dissociates it from the core APC/C resulting in FZR1 inactivation [30,31]. E2F mediated accumulation of cyclin A at the G_1_/S transition also results in phosphorylation of FZR1 [32]. Phosphorylated FZR1 is targeted by SCF E3 ligase, further limiting the activity of APC/C^FZR1^ [33]. In *Caenorhabditis elegans*, cyclin D1/CDK4 phosphorylates N terminus of FZR1 and linker domain of LIN-35, the pRB homologue thereby counteracting the cell cycle inhibitory functions of both the proteins in G_1_ [34]. In *Drosophila* endocycle in which cells undergo repeated rounds of DNA replication with no intervening mitosis, cyclin E/CDK2 mediated phosphorylation of FZR1 drive the periodicity of APC/C^FZR1^ activity [35].

In addition, inhibitory protein Emi1 (also known as FBXO5), not only inhibits APC/C^CDC20^ activity in S- and G_2_-phases but also inhibits APC/C^FZR1^ in interphase by binding like a pseudo-substrate to the APC/C and also by antagonizing the two E2s that function with APC/C^FZR1^ [7,36]. Similarly, the meiotic function of APC/C^FZR1^ is blocked by Emi2, a homologue of Emi1. The levels of Emi1 start increasing at the start of G_1_ and are brought down by SCF^βTRCP1^ in early mitosis to allow activation of APC/C^CDC20^ [37]. Interestingly, Emi1 expression is under the control of E2F which promotes G_1_-S transition when released by phosphorylation of pRB by cyclin/CDK4, 6.

### Consequences of loss of FZR1

A number of studies have investigated the consequences of aberrant *FZR1* expression and its loss on cell cycle and tumorigenicity (Table 2). In budding yeast, FZR1 is required for destruction of mitotic cyclin during mitotic exit [38] but in *Drosophila* and frog embryos it is not required for mitotic exit [39–41]. Major outcomes of *FZR1* knockdown in different mammalian cells due to stabilization of several FZR1 substrates are shortening of G_1_-phase, a premature and prolonged S-phase, delayed entry into mitosis and aberrant chromosomal separation and cytokinesis [42]. Conditional knockout of *FZR1* is lethal in mouse and embryos die at around E10 due to inability of placental trophoblast cells to endoreduplicate. This lethality is prevented when FZR1 is re-expressed in placenta [43]. Recent findings that FZR1 is required for regulation of G_2_/M transition during differentiation of placental trophoblast cells in mice [44], provide an explanation for the previous findings of Garcia-Higuera et al. [43]. Cells derived from *FZR1* knockout mice develop both numeric and structural chromosomal defects indicating that FZR1 is needed for genomic stability [43]. *FZR1* heterozygous mice develop tumours of the mammary gland, lung, kidney, testis, sebaceous glands and B-cell lymphomas [43]. More recent studies with oocyte specific deletion of *FZR1* show that it is not required for completion of meiosis and viable pups could be obtained when *FZR1* negative females were mated with normal males. However, absence of both female and male *FZR1* led to major genomic instability with embryos arrested at first mitotic division [45]. All these studies suggest that FZR1 is essential for maintenance of genomic integrity and its deficiency leads to tumorigenesis. Therefore, FZR1 has been proposed to be a putative haploinsufficient tumour suppressor [24,42,43].

**Table 2:** Consequences of FZR1 depletion in cells and model animals

Cells/model	Effect of FZR1 depletion	Reference
**Yeast**		
Fission yeast *FZR1Δ* gene disruption mutants	*Sterility*, defective in cell cycle arrest in the G_1_-phase upon starvation	[158]
Fission yeast *FZR*1*Δ* gene disruption mutants	Meiotic mutant with aberrant asci having one or two mature spores	[159]
Budding yeast gene replacement *FZR1* mutants	Premature exit from meiotic prophase I	[160,161]
Budding yeast *FZR1Δ* mutants	Inhibition of mitotic cyclin degradation and inappropriately induced DNA replication	[162]
**Mammalian cell lines**		
*FZR1* shRNA treated rat cortical neurons and SH-SY5Y human neuroblastoma cells	Increased proportion of cells in S-phase, apoptosis	[3]
*FZR1* siRNA treated Saos2	Loss of cell cycle arrest, increased generation time	[64]
Lentiviral RNAi mediated KO of *FZR1* in HeLa	Early onset of DNA replication	[42]
Human fibroblast cells	Premature senescence	[46]
MEFs from *FZR1*-KO mice	Poor proliferation, premature senescence	[163]
*FZR1* shRNA treated HeLa	Increase in half-life of SKP2	[164]
*FZR1* shRNA treated HCT 116	Sub-G_1_ DNA content	[164]
***Arabidopsis***		
*Arabidopsis xcm9* mutant with loss of function allele of *FZR1*	Premature termination of floral shoots, disruption of cell cycle progression, defects in *cyclin B1* expression, defects in endoreduplication	[165,166]
***Drosophila***		
Loss of function *Drosophila* mutants of *FZR1*	Reentry into the cell cycle following embryonic cycle 16 thereby bypassing the normal G_1_ arrest	[39]
*RAP/FZR1* loss-of-function mutants of *Drosophila*	Changes in size and morphology of synapses, locomotion defects	[167]
***C. elegans***		
RNAi mediated inactivation of *FZR-1* in *C. elegans*	Sterility, aberrant germ cell proliferation	[168]
**Mouse**		
Conditional knockout mouse	Embryonic lethality at E9.5–E10.5	[43]
*FZR1*^−/+^ mouse	Increased susceptibility to spontaneous tumours	[43]
*FZR1* KO mouse embryos	Embryonic lethality, lack of endoreduplication, placentation defects	[45]
Male *FZR1* germline knockout (KO) mice	Abnormal proliferation of spermatogonia, infertility, failure of early meiotic prophase I in male germ cells	[169]
Female *FZR1* germline KO mice	Premature onset of ovarian failure, subfertile females, defects in early meiotic prophase I	[169]

### FZR1, SKP2 and p27^KIP1^ in human cancers

Normal human fibroblasts undergo premature senescence after acute loss of FZR1, hinting at a built-in fail-safe mechanism against cancer development and the possible underlying molecular mechanism for the less frequently observed FZR1 loss in tumour cells. Thus, it is possible that loss of FZR1 occurs late in tumour development [46]. Nevertheless, SKP2, an FZR1 target, is up-regulated in many cancers [47–49]. SKP2 recruits the cyclin-dependent kinase inhibitory protein (CKI) p27^KIP1^ to the SCF complex for degradation. A variety of carcinoma show a low level of p27^KIP1^ [48,50,51]. Decreased p27^KIP1^ levels are correlated with high grade of malignancy, low survival rate, greater tumour size and histological differentiation suggesting possible role of p27^KIP1^ as a promising prognostic marker for cancer. A number of solid tumours including lung, breast, ovarian, prostate, colon and squamous cell carcinoma manifest conditions of high SKP2 accompanied by low p27^KIP1^considered to be associated with highly aggressive tumours [48,49,51–55]. Human colorectal tumour arrays show higher percentage of SKP2 positive samples and lower percentage of FZR1 and p27^KIP1^ positive samples and high FZR1 expression was associated with tumours showing lower grade histology [56]. These data demonstrate a pathological correlation between FZR1, SKP2 and p27^KIP1^ and suggested that FZR1 levels could be used as a prognostic marker in colorectal samples. Similar investigations in other types of cancers would indicate whether it is applicable to other cancers as well.

Reduced expression of FZR1 is observed in several other tumours other than colon, including brain, liver, ovary, breast and prostate [57] but it is also overexpressed in certain malignant tumours concomitantly with Emi1 [58]. This may represent a compensatory mechanism as overexpression of Emi1 can overcome the cell cycle block due to FZR1 overexpression [59]. It is noteworthy that in these studies levels of APC/C substrate SKP2, securin, aurora A, PLK1, FZR1 and Emi1 correlate positively with malignancy.

## G_1_-S REGULATION BY RETINOBLASTOMA

**Figure 2:**
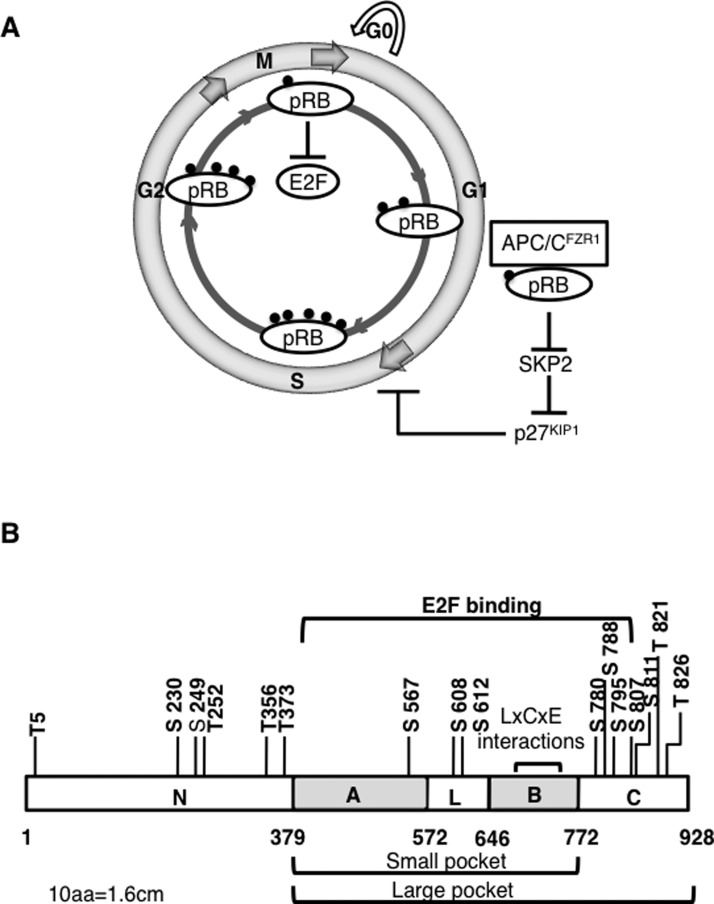
Different mechanisms of G_1_-S regulation by pRB Transcriptional and post-translational regulation of G_1_-S transition by pRB-E2F and pRB-APC/C^FZR1^ complexes. The phosphorylation status of pRB is indicated by black solid balls, with numbers of balls reflecting hypo- or hyper phosphorylation of pRB (not actual number of phosphorylation sites) (**A**). Domain organization of pRB showing phosphorylation sites and regions of interactions with various cellular proteins. Numbers show amino acid positions (**B**).

The tumour suppressor pRB plays a crucial role in not only regulating G_1_ to S transition, but also in quiescence, differentiation and senescence [60]. Its role in inhibiting G_1_-S transition by transcriptional regulation is the best understood among its many different functions. There are two other family members, p107 and p130, that also act as suppressors of cell proliferation by modulating transcription of genes required for cell cycle progression [61]. The canonical model for G_1_-S regulation by pRB is the sequestration of E2F family of transcription factors by hypophosphorylated pRB and release of E2F upon hyperphosphorylation of pRB that promotes the transcription of S-phase genes (Figure 2A) [62]. Although simple and elegant, this model does not explain the retention of tumour suppressor activity of pRB mutants that cannot bind E2F [63].

The search for an alternative mechanism of G_1_ regulation by pRB led to the discovery that APC/C^FZR1^ and SKP2 simultaneously bind to pRB resulting in SKP2 degradation and accumulation of p27^KIP1^ [64]. Wild-type pRB expressing cells can arrest in G_1_ before E2F mediated transcriptional repression, and a pRB mutant R661W, defective for binding to E2F *in vitro*, retains the ability to interfere with SCF mediated degradation of p27^KIP1^ [65]. In the absence of growth-promoting signals, pRB interacts with the N terminus of SKP2 and inhibits SKP2 mediated p27^KIP1^ degradation [65]. The findings of Binne et al. [64], provided a satisfactory explanation of these earlier studies and demonstrated the existence of a post-translational mechanism of pRB with APC/C^FZR1^ that may precede and is distinct from the pRB–E2F mediated transcriptional control for G_1_ arrest (Figure 2A). Apart from SKP2, PLK1 also showed significant accumulation when pRB levels were down-regulated by shRNA whereas other known cell cycle proteins like CDC20, aurora A or geminin were not affected [64]. Although pRB–APC^FZR1^ interaction was not detected in nontransformed, primary human fibroblasts growing asynchronously, it could be detected when these cells were contact inhibited suggesting additional factors controlling the interaction of pRB and FZR1. pRB depletion from contact inhibited U2OS osteosarcoma cell line caused an accumulation of SKP2 suggesting crucial role of this interaction for cellular differentiation compared with proliferation situations [64,66]. Accumulation of other FZR1 substrates like PLK1, but not those of CDC20 substrates, cyclin B1 and securin, in these cells also suggests that there may be other substrates whose levels may be regulated by pRB–FZR1 interaction. Identification of these substrates and what pathways they function in will allow a better understanding of mechanistic aspects of pRB mutations in various cancers.

Genetic investigations reveal that unlike p107 or p130, only pRB mutations are commonly found in human cancers. Studies on mice lacking different combinations of pocket pRB/p107/p130 genes suggest that pRB has significantly stronger tumour suppressor properties than p107 or p130 [61,67]. Retinoblastoma contains three functional domains: The N-terminal domain (RB-N), followed by the AB and the C pockets (Figure 2B). The pRB-AB pocket provides a conserved structural motif called the double cyclin fold found in cyclins which function as modules for protein recognition [68]. There is structural similarity between pRB N-terminal domain and the AB cyclin-like folds suggesting domain duplication [69]. Domains A and B interact with each other along an extended interdomain interface to form the central ‘pocket’ [70,71] which is essential for the tumour-suppressor activity of pRB [72]. Most mutations in pRB are associated with pocket domains. The AB pocket domain is not only crucial for interaction of pRB with E2F but also a target of several transforming viruses as detailed below.

### Inhibition of pRB function by viral oncoproteins

**Figure 3:**
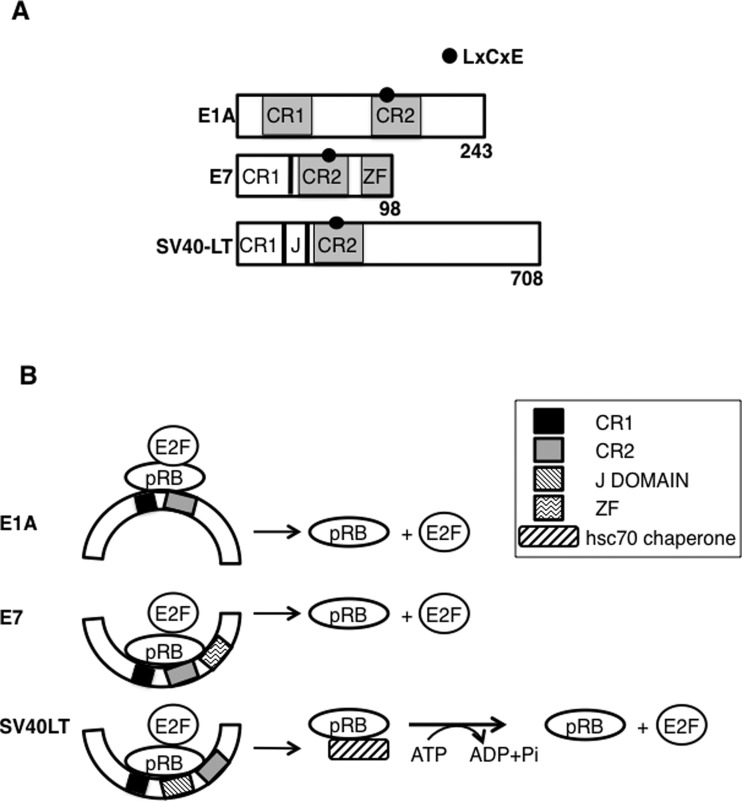
Domain organization of viral proteins LxCxE motif is indicated with solid black ball. Numbers indicate amino acids (**A**). Mechanisms of displacement of E2F from pRB by LxCxE containing viral proteins (**B**).

Transforming viruses like human papilloma virus (HPV), adenovirus and SV40 attack pRB via proteins containing an LxCxE motif (Figure 3A). HPV E7, SV40 large T-antigen (LT) and Adenovirus 5 E1A proteins bind to pocket proteins and displace E2F transcription factors. Like pRB, p130 and p107 are also predicted to contain LxCxE-binding clefts, which bind viral proteins. High risk HPV16 E7 and 48E7 target other pocket proteins like p130 in addition to pRB to overcome cell cycle block. LxCxE motif is a ligand short linear motif (SLiM) that often acts as a simple interface that recruits proteins to multi-protein complexes. This SLiM of viral and cellular proteins binds within a cleft located in the B-pocket of pRB (Figure 2B). The hydrophobic groove in pRB pocket domain B which forms the LxCxE motif binding site consists of four conserved amino acids Tyr-709, Lys-713, Tyr-756 and Asn-757 which are involved in contacting the backbone of the LxCxE peptide. Mutation of these contact amino acids inhibited binding of pRB to LxCxE motif carrying proteins [71,73–75]. Crystal structure of pRB reveals that the region of pRB where the LxCxE peptide binds consists of a patch of positively charged amino acids [71]. A series of E7- and HDAC-1-derived peptides with single or double amino acid substitutions in LxCxE motif was used to show that any positively charged residue in and around the LxCxE peptides had a significant effect on weakening the binding [76].

Although several oncogenic viral proteins display a high affinity binding to pRB via the LxCxE motif, the molecular mechanism of displacement of E2F from pRB is different in each case (Figure 3B). Small DNA viruses such as adenovirus, HPV and polyomavirus carry conserved regions (CRs). The LxCxE motif is found in the CR2 region in these DNA viruses (Figure 3A) [77]. In spite of the fact that these viral oncoproteins share the LxCxE motif, its location is in considerably dissimilar molecular context explaining the differential mechanisms of displacement of E2F by viral LxCxE proteins (Figure 3B). Although CRI of E1A is sufficient to compete with E2F for binding to pRB, the equivalent region of E7 is neither necessary nor sufficient [78–80]. HPV E7 uses high affinity LxCxE mediated binding followed by engagement of E7-C terminal region with pRB C domain which is required to displace pRB–E2F [81] and also destabilizes pRB through proteasomal degradation via reprogramming of the cullin 2 ubiquitin ligase complex [82–84]. CR1 region of Adenoviral-E1A and the transactivation domain (TA) of E2F compete for the same binding site on pRB and thus E1A uses a competitive binding to pRB (Figure 3B) [78]. SV40 LT antigen possess N-terminal J domain that recruits hsc70, a DnaK homologue and uses a chaperone like mechanism to displace pRB–E2F complex. The pRB–E2F complex is then transferred from SV40LT to the ATPase domain of hsc70 resulting in ATP hydrolysis and conformational changes in hsc70 thereby releasing pRB and E2F as separate molecules (Figure 3B) [85]. These results suggest that pRB associates with viral oncoproteins and E2F through overlapping but distinct domains and viral LxCxE is necessary for its binding to pRB, though not sufficient to displace E2F.

The binding kinetics of LxCxE to pRB is modulated by a casein kinase II and PEST sequence (CK2-PEST) present in all three viral proteins HPVE7, SV40LT and AdE1A. It is believed that the complex between LxCxE and pRB is stabilized by CK2-PEST by increasing electrostatic interactions that can aid in a faster kinetics of association [81]. Although the PEST sequence is supposed to contribute to long-range interactions, the phosphate groups contribute to both long and short-range interactions. The long range interactions have fewer conformational restrictions, the short range interactions will be conformationally restricted and therefore are likely to form later during association reaction [81].

Mutations in the LxCxE binding cleft of pRB do not prevent pRB from binding and inactivating E2Fs [86]. Full-length E7 protein carrying mutations outside the LxCxE motif inhibits RB-E2F binding, but retains cell cycle arrest [87], demonstrating that LxCxE dependent interactions of pRB to be distinct from pRB pool binding to E2Fs. These studies also indicate that E2F independent pRB pathways guard proliferative pathways. Biochemical, biophysical and molecular dynamics studies show that the binding of E7 peptide with pRB–E2F complex alone is not sufficient for the dissociation of E2F [88–90] leading to the idea that pharmacological molecules can be designed that can inhibit the binding of E7 without displacing E2F.

### pRB mutations affecting LxCxE based interactions

Many mutations within the pRB LxCxE binding cleft disrupt the interaction of pRB with the viral oncoproteins [73]. An M704V variant of pRB retains its ability to interact with E2F3/DP1, but its ability to interact with LxCxE of SV40LTis greatly compromised [91]. A C706F variant found in small cell lung cancer is unable to interact with LxCxE motif of SV40-LT and adenovirus E1A, whereas retaining its interaction with E2F transcription factors [92,93].

A number of naturally occurring point mutations of pRB found in cancer cells result in disruptions of the integrity of the AB pocket [94,95]. V654L mutation results in reduced penetrance, but substitution of glutamic acid for valine at the same position yield a highly penetrant phenotype [96,97]. Structural studies reveal that this valine residue lie 90–100% buried within the pRB pocket domain. Hence substitution of valine to a charged residue may possibly alter the protein structure [71]. However, effect of this mutation in its binding to E2F and LxCxE has not been determined. Some pRB mutations have extremely low penetrance caused by pRB promoter mutations or splice mutations resulting in truncation of translation of unstable mRNA resulting in reduced pRB expressivity [98]. These weak alleles suppress tumorigenesis in the biallelic state but not in the monoallelic state. These include a deletion involving exons 24 and 25 [99], a splicing mutation C712R at the final base of exon 21 [100], a deletion of Asn-480 and a point mutation R661W [96,101]. Surprisingly, all these pRB mutations reside in the B box of pRB and result in reduced binding to LxCxE proteins and minimal binding to E2F [98,102]. C712R mutation is in proximity to Lys-713, which is a key residue in binding to LxCxE-containing proteins such as HDAC [71,86]. These low penetrant mutants defective in binding to E2Fs and LxCxE, retain partial tumour suppressor activity suggesting that E2F and LxCxE binding are not the only mechanisms through which pRB inhibits cell growth [103,104].

Many cellular proteins that interact with pRB also contain LxCxE motif and many of these proteins are chromatin modifiers [105]. It can be expected that mutations in LxCxE binding region in pRB potentially affect many cellular interactions and defining the molecular basis of any observed phenotype is difficult. Therefore, deletion or point mutation of LxCxE motif of individual pRB interacting proteins in isolation is required to understand the functional importance of this motif in different cellular partners of pRB.

## INTERACTION OF LxCxE CONTAINING CELLULAR PROTEINS WITH pRB

Approximately 30 cellular proteins, including RBP1, RBP-2, CtIP, EID-1, MAP3K5 (ASK-1), gankyrin, cyclin D1, D2, D3 and HDAC-1 and -2, that bind to pRB have a conserved LxCxE motif. However, mutations in the LxCxE motif of cellular proteins have varied effects on interaction and function with pRB (Table 3). Mutations in LxCxE motif of cyclin D1, D2 and ASK-1 profoundly diminish their binding to pRB. The LxCxE motif of cyclin D1 is not necessary for its function, but the motif is required by cyclin D2 [106–108]. Although mutation in LxCxE motif of RBP-1 replacing ‘E’ to ‘K’ retains its binding to pRB [109], mutation at similar position replacing ‘E’ to ‘A’ in oncoprotein gankyrin abrogates its binding to pRB. Gankyrin binding to pRB accelerates phosphorylation and degradation of pRB, releasing E2F transcription factor to activate DNA synthesis genes [110]. Surprisingly, mutation within the LxCxE motif of RBP2 resulted in loss of ability to precipitate p107, whereas retaining its binding to pRB [109]. Replacement of LxCxE of BRCA1 to RxRxH retained its binding to pRB. However, RxRxH mutation altered the tumour suppressor activity of BRCA1 [111]. These studies suggest that the mere presence of the motif is not predictive of its importance for interaction and function of pRB.

**Table 3:** Effects of mutations in the LxCxE motif of cellular proteins on their function and interaction with pRB

pRB binding protein	LxCxE mutation	Effect on binding to pRB	Effect on function	References
RBP-1	E to K	Retains binding to pRB	ND	[109]
HBP-1	C to G in both LxCxE and LxCxE	No binding to pRB	Loss of transcriptional repression by HBPl	[170]
CtIP	Deletion	No binding to pRB	ND	[171]
HDAC-1	Deletion	No binding to pRB	Inhibition of pRB repressive function	[172,173]
BRG-1	Mutation in pRB LxCxE cleft	Retains binding to pRB	ND	[86]
EID-1	Deletion	No binding to pRB	No effect	[174]
Gankyrin	E to A	No binding to pRB	Inhibition of phosphorylation and degradation of pRB	[110]
RFC-1	C to G, E to K	No binding to pRB	Loss of RFC function in promoting cell survival after DNA damage	[175]
BRCA-1	RXRXH	Retains binding to pRB	Altered tumour suppressor activity of BRCA1	[111,172]
ASK-1	LKCFE to VRCFD	No binding to pRB	Altered ASK-1 activity in induction of apoptosis	[106]
Cyclin D1, D2	C to G, E to K	Profoundly diminished binding to pRB	Partial abrogation of pRB-induced growth arrest and senescence	[107,176]
RBBP-9	L to Q	No binding to pRB	ND	[177]
NuMA	C to G, E to K	No binding to pRB	Abnormal organization of spindle microtubules	[178]

Unlike HPVE7, SV40 LT and AdE1A, that all have the CK2-PEST sequences, only some cellular LxCxE containing proteins have an acidic region or phosphorylation site whereas others do not. No systematic study has been carried out to correlate the presence or absence of such regions on modulation of binding of LxCxE containing cellular proteins with pRB. Viral oncoproteins carrying LxCxE motif can displace cellular proteins like cyclin D, HDAC-1 and BRG1 from pRB [112]. Cyclin D and BRG1 do not have an acidic region whereas HDAC-1 has an acidic region. Thus, presence or absence of acidic region does not seem to determine whether viral proteins can displace cellular proteins from pRB. Rather, binding affinities and phosphorylation status may be more important.

pRB is a conformationally plastic protein in response to phosphorylation [113]. Among the 16 phosphorylation sites identified, no phosphorylation site is found in the B-pocket suggesting that modifications in this structure are not tolerated [114]. Phosphorylation at Thr-821 and Thr-826 inhibits binding of pRB B-box to LxCxE motif containing proteins without affecting pRB binding to E2Fs [113]. The AB domain is reported to be metastable whose native state can be destabilized even by mild perturbations [115]. RB-AB unfolding presents a three-state transition involving cooperative secondary and tertiary structure changes and a partially folded intermediate that can oligomerize. This property of pRB-AB may allow the formation of multi protein complexes, constituting a robust mechanism to retain its cell cycle regulatory role [115]. Equilibrium unfolding studies suggest that the equilibrium between native and non-native states of the AB domain is strongly influenced by LxCxE and other ligands, with degree of stabilization correlating with ligand binding free energy. Molecular dynamics studies suggest that the stability of pRB-AB in the apo- and in E7 bound forms is similar but it increases in the E2F bound form. The binding of E7 peptide through its LxCxE motif with the B box of pRB induces the conformational changes around A–B interface where E2F binds to pRB [115]. These studies suggest that pRB native structure is stabilized *in vivo* by interactions with its numerous ligands and the native state may be very sensitive to destabilization by mutations. There are many mutations reported for pRB in various databases of cancers but the mechanism by which they affect pRB function is not understood in each case. Although some of the missense mutations may have no effect, others may affect the stability and conformational dynamics of pRB and its ability to interact with LxCxE containing proteins that can compromise its function.

### pRB–APC/C^FZR1^ interaction

Although there is lack of structural details of interactions between APC/C and pRB, studies on RB mutagenesis suggest that FZR1 binding involves the pRB LxCxE-binding cleft in the AB pocket [64]. Unlike E2Fs and viral oncoproteins, the possibility that the same RB molecule can regulate E2F and APC–SKP2–p27^KIP1^ activities simultaneously does not seem plausible. It is also unclear whether pRB–SKP2–p27^KIP1^ pathway is equivalent in importance with E2F transcriptional repression in cell cycle regulation. Interestingly, neither p107 nor p130 display any affinity for APC/C subunits [64]. Further, pRB associates exclusively with FZR1 and not CDC20 [64]. The C-terminus WD40 domain of FZR1 contains a motif similar to the LxCxE motif. Loss of the C-terminal 100 amino acids of FZR1 impairs pRB binding [64]. The presence of LxCxE motif within this deleted region hints at a possible role of this motif in binding of FZR1 to pRB but the importance of the motif for interaction with pRB is not known. HPV E7 protein bound to pRB establish a dual-contact mode with 90% of the binding energy determined by the E7 LxCxE motif, with an additional binding determinant located in the C-terminal domain of E7 [90]. Whether FZR1 has a single or multiple contact sites for pRB is not known. The modular structure of pRB and the fact that both pRB and FZR1 interact with multiple partners, binding between FZR1 and pRB is likely to be weaker compared with HPV E7 peptide to allow the periodic changes in the downstream targets like SKP2 that are required for normal cell cycle.

*In vitro* studies show that FZR1 can regulate E2F activity in G_1_ that involves its interaction with hypophosphorylated pRB but independent from its interaction with APC/C [46]. Another example of APC/C independent function of FZR1 is promotion of Smurf1 E3 activity *in vitro* by a C-box deletion mutant of FZR1 that cannot bind to the APC/C core implicating it in osteoblast differentiation [116], although pRB interaction was not checked in this study. If confirmed *in vivo*, these results show that there may be APC/C independent functions of FZR1–pRB other than cell cycle regulation and should be taken into consideration while designing any inhibitor or drug against these target proteins.

### APC/C and pRB as drug targets

It is becoming clear in recent years that the role of proteins defined as tumour suppressors is not as clear cut as previously thought and the cellular and genetic context defines the functional outcome of mutations, inactivation and dosage effects. Both APC/C and pRB regulate many, and often contradictory functions in a cell and decide the fate of many proteins. Both are also targets of various transforming viruses. During cancer progression, changes in developmental phenotype are thought to involve pRB [117,118]. Due to their many interacting partners and the fact that APC/C is a multi-subunit assembly where the functions of each subunit are still not defined, both protein interface drug discovery and high-throughput screening approaches may need to be explored. The latter has been used to find promising lead compounds inhibiting APC/C and SKP2/CKS1 interaction [119,120].

Various components of the UPP are considered attractive drug targets and the success of Bortezomib, a proteasome inhibitor, in treatment of multiple myeloma has pushed UPP at the forefront as drug target. Of the three classes of enzymes in the pathway, E3s are considered to be better targets compared with E1 and E2 because they determine the substrate specificity thus potentially more targeted therapies can be developed. Conversely, multiplicity of substrates can be a challenge for targeted therapies. The other concerns are the therapeutic window and the selectivity between normal and cancer cells. Despite of these concerns, the attractive feature of APC/C as a potent drug target is that outcome of APC/C activity can be controlled by modulating either of the two adaptor proteins–FZR1 or CDC20. Anti-mitotic reagents like Taxol and Nocodazole, used as anti-cancer therapies, activate the SAC presumably through inhibiting APC^CDC20^ [121]. Therefore, development of inhibitors that will specifically target CDC20 could reduce the off-target and side effects. CDC20 is a preferred target compared with FZR1 as its function and substrates are mostly restricted to mitosis whereas FZR1 has a much broader substrate range and functions. However, due to its role in maintenance of G_0_/G_1_ state and inhibition of G_1_-S transition along with pRB, agonists can perhaps be designed that can promote these functions of FZR1. For example small molecules that may stabilize FZR1–pRB interaction or inhibit the release of FZR1 from pRB may prove useful to prevent proliferation. Interdependence between FZR1, SCF, CDC20 and Emi1 can also be exploited by combining antagonists and agonists [122]. Centrosome amplification, a common feature of many cancer cells has been proposed to drive genomic instability. Degradation of the motor protein kinesin-5 (Eg5) by APC/C^FZR1^ is required for the clustering of these extra centrosomes. Accumulation of Eg5 in cells expressing FZR1 carrying mutations in certain D- or KEN-box motifs causes cancer cell death [123]. These studies hint future prospects of developing drugs targeting FZR1 at specific motifs.

The strategies for drug development with pRB as a target may include pRB-mediated promotion of cell cycle inhibition, senescence, apoptosis or differentiation of cancer cells or exploiting its absence for targeted killing. Each of these strategies will have to take into account the status of pRB in different cancers. Structurally defining individual protein interaction surfaces within or outside the pocket domain of pRB that mediate some of the pRB-specific tumour suppressor functions and that are not conserved in p107 and p130 represent attractive drug targets for pRB. Cells deficient in pRB are more susceptible to apoptosis induced by DNA damage and this capability is linked to its growth suppression activity. This property is useful for cancers that are pRB negative. Indeed, pRB negative breast cancer cells are more sensitive to chemotherapy compared with pRB positive breast cancer cells [124]. Since cyclin D/CDK4, 6 mediated phosphorylation of pRB results in release of growth promoting E2F, CDK inhibition has been explored as a strategy to prevent pRB inactivation and reestablish cell cycle control. Among these inhibitors flavopiridol and roscovitine have a very broad range of targets but can inhibit cell cycle and cause cell death. Despite this flavopiridol is not effective in many cancers [125]. More specific inhibitors have been developed against CDK4/6 among which palbociclib, the most successful, has been evaluated in mono- as well as combination therapy and is in the Phase III trial [126]. However, a range of sensitivities to palbociclib was noted in breast cancer [127].

Since the binding of E7 LxCxE peptide with pRB–E2F complex alone is not sufficient for the dissociation of E2F, pharmacological molecules can be designed that can inhibit the binding of LxCxE dependent interactions of viral proteins with pRB without disturbing pRB/E2F interactions [88]. However, such an approach will need to consider the effect on cellular LxCxE containing proteins. Perhaps a better strategy will be to exploit phosphorylation mediated plasticity of pRB to prevent its inactivation. This approach has been used to investigate the efficacy of an LxCxE peptide as an inhibitor of phosphorylated T373 mediated conformational change that weakens the interaction of E2F with pRB [128]. The LxCxE peptide from HPV E7 was found to have a modest effect and full length HPV E7 had better effect on activation of phosphorylated pRB *in vitro* in this study. Significantly, LxCxE peptides derived from cyclin D could not activate pRB.

## FUTURE DIRECTIONS AND PROSPECTS

Both APC/C and pRB are critical determinants of important cellular decisions regarding proliferation, differentiation and quiescence. Both are also targeted by several viruses for proliferative advantages. The interaction of these two important players adds another layer to the G_1_-S regulation which is an important phase of the cell cycle in which cells can choose between different fates. This interaction, although providing an explanation for the retention of growth suppressor activity of pRB mutants defective in E2F binding, opens many important new questions. Further research is required to understand the mechanisms that regulate the FZR1–pRB interaction, the number and type of APC/C^FZR1^ substrates that are affected by this interaction, whether pRB makes contacts with other APC/C subunits and the molecular mechanism by which pRB stimulates SKP2 ubiquitination. What other substrates, other than PLK1 and SKP2 are directly affected by this interaction is also an open question. Answers to these questions will provide further insight into the molecular mechanism of APC/C function and provide clues that can be exploited to develop better inhibitors.

It is interesting that among cellular proteins carrying LxCxE motif that bind to pRB, several are ubiquitin ligases. Apart from APC/C^FZR1^, BRCA-1 and NRBE3 are other E3 ligases that bind to pRB. NRBE3 is a novel pRB ligase that promotes pRB degradation and is transcriptionally regulated by E2F transcription factors [129]. All the three E3 ligases contain LxCxE motif (Ramanujan and Tiwari, unpublished observations). Future work is required to functionally and structurally dissect the role of ubiquitin ligases in association with pRB and to identify E3 ubiquitin ligases responsible for proteasome mediated degradation of pRB. There are increasing evidences of viruses inactivating key cell cycle players, the most recent being that of Epstein–Barr virus (EBV) protein kinase BGLF4 directly binding and phosphorylating CDC20, possibly allowing CDC20 to be targeted by SCF for degradation [130]. EBV nuclear antigen 3C (EBNA3C) is also linked to regulation of the SCF complex thereby mediating degradation of pRB through the SCF^SKP2^ complex [131]. We speculate that transforming viruses might disable APC/C throughout the cell cycle, by inactivating its activity mediated by both its adaptor proteins, FZR1 and CDC20. It is important to understand the mechanistic basis of these interactions and understand their functional implications to be able to develop better anti-viral and anti-cancer drugs.

## Abbreviations

APC/C: anaphase promoting complex/cyclosome
CK2-PEST: casein kinase II and PEST sequence
CKI: cyclin-dependent kinase inhibitory protein
CR: conserved region
E1: ubiquitin-activating enzyme
E2: ubiquitin-conjugating enzyme
E3: ubiquitin ligase
EBV: Epstein–Barr virus
HPV: human papilloma virus
LT: large T-antigen
PLK1: Polo-like kinase-1
pRB: retinoblastoma protein
SAC: spindle assembly checkpoint
SCF: Skp1/Cul1/F-box
SLiM: short linear motif
Ub: ubiquitin
UPP: ubiquitin proteasome pathway
